# Children of parents with alcohol problems: life trajectories, professional development, and coping processes. A qualitative study with professionals and students in the addictions field

**DOI:** 10.1192/j.eurpsy.2026.10503

**Published:** 2026-07-31

**Authors:** F. Calvo, M. Buera-Prius, M. Pallisera

**Affiliations:** Departament de Pedagogia, Universitat de Girona, Girona, Spain

## Abstract

**Introduction:**

Children of parents with alcohol use disorders (AUD) are at higher risk of adverse childhood experiences (ACEs), emotional neglect, and relational difficulties. Yet, research also highlights the development of psychosocial skills and resilience derived from these contexts. While international evidence is growing，empirical studies in Spain remain scarce, particularly regarding the dual impact—vulnerability and strength—on adult trajectories and professional practice.

**Objectives:**

To explore life experiences of adults who grew up in families with alcohol problems.

To analyze perceived competencies and limitations in personal and professional development.

To identify coping and self-care strategies across their trajectories.

**Methods:**

A qualitative design was applied. Three focus groups (n=16) were conducted with professionals in addiction services and students in a master’s program on addictions, all of whom had at least one parent with alcohol problems. Transcripts were coded through combined structural and descriptive coding, followed by thematic analysis. Categories were triangulated among researchers and contrasted with findings from an international systematic review. Ethical approval and informed consent were obtained.

**Results:**

Ten main categories emerged: (i) adverse childhood experiences (role reversal, secrecy, emotional neglect); (ii) acquired competencies (empathy, anticipation, conflict management, precocious maturity); (iii) personal limitations (self-blame, perfectionism, shame, difficulty valuing achievements); (iv) professional facilitators and barriers (emotional attunement, difficulties setting boundaries); (v) influence on career choice toward helping professions; (vi) coping mechanisms (sport, writing, nature, volunteering); (vii) hypervigilance and control dynamics. Participants described a persistent tension between emotional overload and a strong vocational drive to help others. Narratives emphasized both the weight of intergenerational trauma and the transformative potential of therapy, education, and social support.

**Conclusions:**

Growing up with parental alcohol problems shapes both vulnerabilities and strengths that significantly influence adult life and professional development. These trajectories reveal heightened empathy, responsibility, and conflict management skills, but also overexertion, boundary-setting difficulties, and impostor feelings. Clinical and educational practice should acknowledge this dual impact, offering tailored psychological support, structured self-care strategies, and validation spaces for professionals with such backgrounds. Incorporating the lived experience of adult children of parents with AUD into training and clinical settings may enhance prevention, early detection, and mental health outcomes in the addictions field.

**Disclosure of Interest:**

None Declared

